# Complete mitochondrial genomes of two marine monogonont rotifer *Brachionus manjavacas* strains

**DOI:** 10.1080/23802359.2021.1935349

**Published:** 2021-06-07

**Authors:** Min-Sub Kim, Young Hwan Lee, Duck-Hyun Kim, Hee-Jin Kim, Un-Ki Hwang, Russell Shiel, Atsushi Hagiwara, Jae-Seong Lee

**Affiliations:** a Department of Biological Sciences, College of Science, Sungkyunkwan University, Suwon, South Korea; b Institute of Integrated Science and Technology, Nagasaki University, Nagasaki, Japan; c Marine Ecological Risk Assessment Center, West Sea Fisheries Research Institute, National Institute of Fisheries Science, Incheon, South Korea; d School of Biological Sciences, University of Adelaide, Adelaide, Australia; e Organization for Marine Science and Technology, Nagasaki University, Nagasaki, Japan

**Keywords:** Monogonont rotifer, complete mitochondrial genome, *Brachionus manjavacas*, Australian strain, German strain

## Abstract

The complete mitochondrial genomes of *Brachionus manjavacas* German strain were 10,721 bp (mitochondrial DNA I) and 12,274 bp (mitochondrial DNA II) in size, while the complete mitochondrial genomes of *B. manjavacas* Australian strain were 10,889 bp (mitochondrial DNA I) and 12,443 bp (mitochondrial DNA II) in size. Of 12 protein-coding genes (PCGs), 99.6% of amino acid sequences were identical between the two strains. Of 12 PCGs of both *B. manjavacas* strains, three genes (*ND1*, *ATP6*, and *ND5*) had incomplete stop codon T. Furthermore, ATA was the start codon for *ND4*, *ND5*, and *CO3* genes, whereas that for other PCGs was ATG in both strains. The base compositions of 12 PCGs in the *B. manjavacas* strains were similar, indicating that the mitochondrial genome of the two strains was structurally conserved over evolution. The gene structure and its orientation of 12 PGCs of *B. manjavacas* strains were identical, as shown in other marine *Brachionus* rotifers and the freshwater *Brachionus* rotifers, while the freshwater rotifer *B. calyciflorus* had an additional cytochrome b gene in the mitochondrial DNA I.

The marine rotifer *Brachionus plicatilis* species complex including *Brachionus manjavacas* (Fontaneto et al. 2007) consists of at least 15 species (Mills et al. 2017). However, there is no report on complete mitochondrial genome of *B. manjavacas*, while several complete mitochondrial genomes of other marine *Brachionus* rotifers have been published, including those of *B. plicatilis*, *B. koreanus*, *B. rotundiformis*, and *B. paranguensis* (Suga et al. 2008; Hwang et al. 2014; Kim et al. 2017; Choi et al. 2020). Thus, construction of a phylogenetic tree of the *Brachionus* rotifers with 12 protein-coding genes (PCGs) of the mitochondrial genome is important to unravel the relationship of species-specificity within the *B. plicatilis* species complex clade. In this study, we identified two complete mitochondrial genomes of the two strains *B. manjavacas* Germany and Australia.

The resting eggs of *B. manjavacas* German strain were collected by Dr. Harald Rosenthal by netting in Schlei-Fjord (54°36′N and 9°51′E) in 1988 and cultured in Nagasaki University in Japan (Fu et al. 1991), while the resting eggs of *B. manjavacas* Australian strain were collected by Dr. Russell Shiel (University of Adelaide) by netting from sediments of Lake Colongulac (38°10′41.6″S and 143°10′52.9″E), West Victoria in 2000. These eggs hatched and were cultured in Nagasaki University, Japan (Kotani et al. 2006). To identify the complete mitochondrial DNA of two strains of *B. manjavacas*, live samples were sent to South Korea. The genomic DNAs obtained from the two strains of *B. manjavacas* (268.2 μm in length and 184.7 μm in width for German strain and 251.6 μm in length and 183.9 μm in width for Australian strain) were deposited at the Ichthyological Collection of the Faculty of Fisheries, Nagasaki University (FFNU) under accession nos. FFNU-Rot-0007 and FFNU-Rot-0008.

We sequenced whole body genomic DNA of the two strains of *B. manjavacas* using the 300 bp paired-end sequencing by NovaSeq 6000 (Illumina, San Diego, CA). *De novo* assembly was conducted by Spades version 3.13.0 (http://cab.spbu.ru/software/spades/). From the assembled *B. manjavacas* genome, 165,113 scaffolds (total 143,544,257 bp; N50 = 12,642 bp) for the German strain and 72,526 scaffolds (total 120,419,270 bp; N50 = 18,615 bp) for the Australian strain, we identified 16 (for German strain) and 9 (for Australian strain) mitochondrial scaffolds and obtained two complete mitochondrial DNA sequences for each strain through manual editing.

The complete mitochondrial genomes of *B. manjavacas* German strain were 10,721 bp (mitochondrial DNA I; GenBank no. MW559988) and 12,274 bp (mitochondrial DNA II; GenBank no. MW559989) in size. Of 12 PCGs, the identity and positives in amino acid sequences between strains were 99.6% and 99.7%, respectively. Three genes (*ND1*, *ATP6*, and *ND5*) of *B. manjavacas* German strain had incomplete stop codon T. Furthermore, ATA was the start codon for *ND4*, *ND5*, and *CO3* genes, whereas the start codon for other PCGs was ATG. The base composition of 12 PCGs in the mitogenome of *B. manjavacas* German strain showed 25.5% for A, 42.6% for T, 18.7% for C, and 13.2% for G. The mitochondrial genome A + T base composition (68.1%) of 12 PCGs was higher than that of G + C (31.9%), as shown in other *Brachionus* rotifers.

Additionally, the complete mitochondrial genomes of *B. manjavacas* Australian strain were 10,889 bp (mitochondrial DNA I; GenBank no. MW559986) and 12,443 bp (mitochondrial DNA II; GenBank no. MW559987) in size. Of 12 PCGs, three genes (*ND1*, *ATP6*, and *ND5*) of *B. manjavacas* German strain had incomplete stop codon T. Furthermore, ATA was the start codon for *ND4*, *ND5*, and *CO3* genes, whereas the start codon for other PCGs was ATG, as shown in *B. manjavacas* German strain. The base composition of 12 PCGs in the mitogenome of *B. manjavacas* Australian strain showed 25.5% for A, 42.6% for T, 18.8% for C, and 13.1% for G, respectively, indicating marginal differences between the two strains of *B. manjavacas*. However, the base composition of A + T (68.1%) and G + C (31.9%) of 12 PCGs was the same as that of the German strain. The gene structure and orientation of 12 PCGs of the *B. manjavacas* strains were identical to those of other marine *Brachionus* rotifers and the freshwater *Brachionus* rotifers, except for the freshwater rotifer *B. calyciflorus* with an additional cytochrome b gene in the mitochondrial DNA I.

The placement of the two strains of *B. manjavacas* in the genus *Brachionus* with 12 PCGs is shown in Figure 1. The two strains were clustered with *B. paraguensis* and *B. plicatilis* (NH1L strain), which are euryhaline species, but was separated from the super small (SS) type of marine rotifer *B. rotundiformis* (Indonesian strain) and the small-medium (SM) type of marine rotifer *B. koreanus* (South Korean strain), possibly supporting the phylogeny with *CO1* +* ITS1* sequences and morphotypes recently reported by Mills et al. (2017).

**Figure 1.**
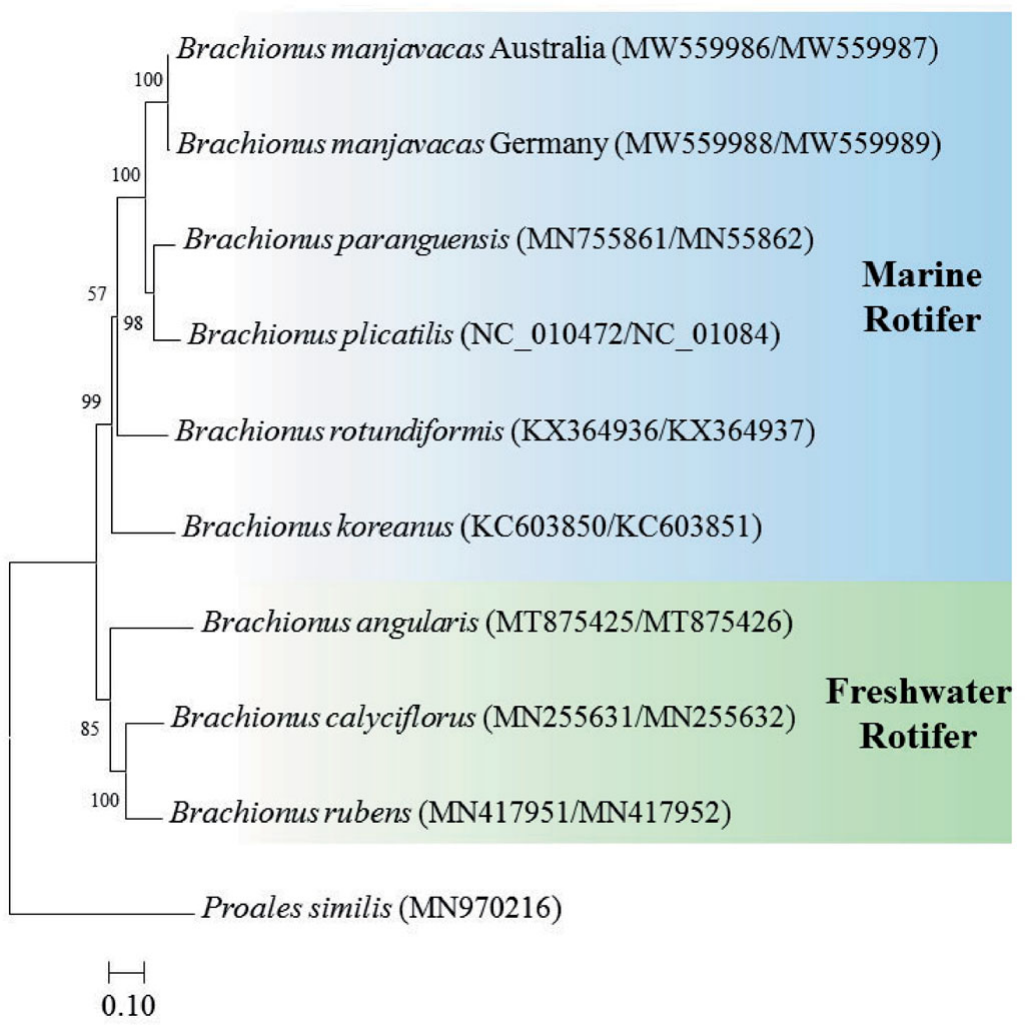
Phylogenetic analyses based on mitochondrial DNA of two strains of *Brachionus manjavcas* with seven *Brachionus* species. The amino acid sequences of 12 mitochondrial DNA genes were aligned by ClustalW. Maximum likelihood analysis was performed by Mega software version 10.0.1 with the Gamma + LG + I model. Rapid bootstrap analysis was conducted with 1000 replications with 48 threads running in parallel. The rotifer *Proales similis* (class Monogononta) served as the outgroup. –Ln = 30,004.91.

## Data Availability

The genome sequence data that support the findings of this study for *B. manjavacas* Australian strain are openly available in GenBank of NCBI at https://www.ncbi.nlm.nih.gov/nuccore under the accession no. MW559986-MW559987. The associated BioProject, SRA, and Bio-Sample numbers are PRJNA716452, SRR14040032, and SAMN18436264, respectively. The genome sequence data that support the findings of this study for *B. manjavacas* German strain are openly available in GenBank of NCBI at https://www.ncbi.nlm.nih.gov/nuccore under the accession no. MW559988-MW559989. The associated BioProject, SRA, and Bio-Sample numbers are PRJNA716453, SRR14040033, and SAMN18436265, respectively.
